# Global Metabolomic Analysis of Human Saliva and Plasma from Healthy and Diabetic Subjects, with and without Periodontal Disease

**DOI:** 10.1371/journal.pone.0105181

**Published:** 2014-08-18

**Authors:** Virginia M. Barnes, Adam D. Kennedy, Fotinos Panagakos, William Devizio, Harsh M. Trivedi, Thomas Jönsson, Lining Guo, Shannon Cervi, Frank A. Scannapieco

**Affiliations:** 1 Colgate Palmolive Technology Center, Piscataway, NJ, United States of America; 2 Metabolon, Durham, NC, United States of America; 3 Department of Oral Biology, School of Dental Medicine, University at Buffalo, State University of New York, Buffalo, NY, United States of America; University of Florida, United States of America

## Abstract

Recent studies suggest that periodontal disease and type 2 diabetes mellitus are bi-directionally associated. Identification of a molecular signature for periodontitis using unbiased metabolic profiling could allow identification of biomarkers to assist in the diagnosis and monitoring of both diabetes and periodontal disease. This cross-sectional study identified plasma and salivary metabolic products associated with periodontitis and/or diabetes in order to discover biomarkers that may differentiate or demonstrate an interaction of these diseases. Saliva and plasma samples were analyzed from 161 diabetic and non-diabetic human subjects with a healthy periodontium, gingivitis and periodontitis. Metabolite profiling was performed using Metabolon's platform technology. A total of 772 metabolites were found in plasma and 475 in saliva. Diabetics had significantly higher levels of glucose and α-hydroxybutyrate, the established markers of diabetes, for all periodontal groups of subjects. Comparison of healthy, gingivitis and periodontitis saliva samples within the non-diabetic group confirmed findings from previous studies that included increased levels of markers of cellular energetic stress, increased purine degradation and glutathione metabolism through increased levels of oxidized glutathione and cysteine-glutathione disulfide, markers of oxidative stress, including increased purine degradation metabolites (e.g. guanosine and inosine), increased amino acid levels suggesting protein degradation, and increased ω-3 (docosapentaenoate) and ω-6 fatty acid (linoleate and arachidonate) signatures. Differences in saliva between diabetic and non-diabetic cohorts showed altered signatures of carbohydrate, lipid and oxidative stress exist in the diabetic samples. Global untargeted metabolic profiling of human saliva in diabetics replicated the metabolite signature of periodontal disease progression in non-diabetic patients and revealed unique metabolic signatures associated with periodontal disease in diabetics. The metabolites identified in this study that discriminated the periodontal groups may be useful for developing diagnostics and therapeutics tailored to the diabetic population.

## Introduction

Periodontal disease is a chronic bacterial infection causing persistent gingival inflammation, and in some cases connective tissue destruction and bone resorption around the teeth. It is also characterized by pocket formation and recession. Although advances in dental care has resulted in improved periodontal status in certain populations, disparities persist as severe periodontitis is often found to be exaggerated in certain segments of the population, for example those from a low socio-economic background [1]. It is well known that bacteria colonize the teeth to form a biofilm, called dental plaque, which initiates gingivitis, and sometimes progresses to periodontitis. Release of bacterial products from the biofilm induces local inflammation. Without treatment, periodontal tissue destruction, bone resorption and tooth loss may ensue [1]. Periodontal disease also has been associated with several systemic diseases, including cardiovascular disease, diabetes mellitus, respiratory disease, rheumatoid arthritis, chronic kidney disease, and adverse pregnancy outcomes [2]–[6].

The gingival epithelium forms a crevice around each tooth that provides a protected space for bacterial colonization and proliferation [7]–[10]. Analysis of gingival crevicular fluid (GCF) [11] and saliva [12]–[14] from periodontal patients has identified a variety of inflammatory mediators and tissue-destructive molecules, including metalloproteinases [15]–[23] and metabolic signatures associated with host-bacterial interactions to be elevated when compared to periodontally health patients.

Diabetic patients have a high prevalence of gingivitis, periodontitis, oral candidiasis, and xerostomia, and the severity of these diseases are correlated with the duration of diabetes and degree of glycemic control [24], [25]. Poor glycemic control has been shown to be associated with poor periodontal health, of which the molecular signatures may be monitored using modern “omics”-based methods [26]–[30].

Saliva is a complex secretory fluid that contains trace metals, metabolites, biochemicals, proteins, glycoproteins, lipds, etc., that serve a spectrum of physiological needs. Saliva is a critical source of tissue lubricants, tooth mineralizing factors, acid buffers, toxin neutralizers, and antimicrobial components [31]–[35]. The ability to use saliva to evaluate physiological conditions, follow disease progression, and monitor post-treatment therapeutic results through noninvasive methods is an important objective for healthcare in general and periodontology in particular. We have previously performed a series of untargeted global metabolomic profiling tests of GCF and saliva samples from subjects with healthy gingiva, gingivitis, and periodontitis that have suggested a rigorous set of potential biomarkers for monitoring periodontal disease status and examining the effectiveness of oral care treatment that resolves the metabolic signature of inflammation [11], [12], [36], [37]. Many metabolites associated with inflammation, oxidative stress, tissue degradation, and bacterial metabolism were found to be significantly elevated in periodontal disease and reduced by treatment [37]. Validation of such biomarkers will provide an objective phenotype to allow practitioners to diagnose disease, monitor patient disease activity and determine the effectiveness of treatment.

Metabolomic profiling is a rapidly evolving technology that has been increasingly used to discover early markers of disease [11], [12], [37]–[40]. Whole saliva can be easily collected through noninvasive means and has considerable potential to monitor general health and disease status. With the advancement of technological means such as metabolomics, saliva can be leveraged as a clinical and diagnostic tool because of its potential to mirror oral and systemic health conditions [41]–[45]. To this end, metabolomic signatures were determined in saliva and compared to those in plasma from orally healthy subjects and periodontal subjects, with and without type 2 diabetes, in an effort to identify unique metabolomic profiles and biomarkers of periodontal disease in both systemic health and diabetes. Based on our previous results from biomarker identification of periodontal disease progression [11], [12], [37], we performed a discovery study to identify biomarkers of decreased periodontal health as monitored by biochemical profiling of saliva and plasma collected from orally healthy subjects and those with gingivitis and periodontitis using an unbiased metabolomic profiling approach based on liquid and gas chromatography/mass spectrometry (LC/MS and GC/MS).

## Materials, Participants, and Methods

### Experimental Design and Participants

This cross-sectional clinical study enrolled adult male and female subjects from the Buffalo, New York area and was conducted in accordance with the Helsinki Declaration. The Institutional Review Board at the University at Buffalo approved the protocol (ORB0650111E), including the clinical examination, collection of saliva and plasma samples. All subjects read and signed a written informed consent before their enrollment into the study. The inclusion criteria were: age between 18 and 65 years, in good general health (n = 81), or subjects diagnosed with diabetes (n = 80); and a minimum of 20 natural teeth (excluding third molars) present. Exclusion criteria included: inability or unwillingness to sign the informed consent form, diagnosis of a medical condition which required pre-medication prior to dental visits/procedures, 5 or more decayed untreated teeth at screening (cavities), diagnosis of other diseases of the hard or soft oral tissues, impaired salivary function, use of antibiotics or antimicrobial drugs within 30 days prior to the first study visit, a history of uncharacterized systemic disease, pregnant or nursing women, participation in any other clinical study within 1 week prior to enrollment into this study, present use of tobacco products, subjects who required dental treatment during the duration of the study, immune compromised individuals (e.g. those diagnosed with HIV, AIDS, or taking immunosuppressive drug therapy), or subjects who received periodontal treatment within the previous 30 days.

Subjects were evaluated by a single dental examiner (SC) for inclusion/exclusion criteria. At the first visit, participants received a tube of standard, non-anti-microbial fluoridated toothpaste with instructions for use, in order to minimize variations in results that might be driven by the use of different toothpastes. A fasting blood sample was then collected from subjects self-identifying as diabetic to confirm diabetic status. During the second visit, subjects gave an update on their medical and medication history, contributed saliva and blood samples, had an assessment of their oral tissues to assess periodontal status, to monitor adverse events since the first visit and answer questions regarding their compliance as to the use of the standard toothpaste. A minimum of 0.5 ml of unstimulated saliva and 10 ml of blood was collected. Subjects were asked to refrain from eating or drinking (excluding water) from 11:00 PM the previous night; to brush their teeth and entire mouth the evening before the saliva donation, but not the morning of collection. Blood samples were obtained after collection of the saliva. Saliva was collected into sterile polypropylene tubes and immediately frozen in a dry ice bath and stored at −80 degrees C until shipped to Metabolon. No preservatives were added to the saliva samples

### Definition of oral status groups

Within the diabetic or non-diabetic cohorts, three sub-groups were established: periodontal health, gingivitis and periodontitis. Subjects were stratified according to the gingivitis index: Healthy: These subjects had an average full mouth Modified Gingival Index (MGI) [46] score below 1.0, with fewer than 3 bleeding on probing (BOP) sites and minimal dental plaque present as scored by the method of Sillness and Lőe [47]. Gingivitis: These subjects had an average full mouth MGI score between 1 and 2 with multiple BOP sites and periodontal pockets <4 mm. Periodontitis: These subjects had an average full mouth MGI of 2.0 or greater with multiple BOP sites, and 2 or more periodontal pockets with probing depths of 5 mm or more in at least two quadrants. Plaque at any level was present. Measurement of oral indices was performed as per Lobene et al. [46] as follows: 0 = normal, 1 = mild inflammation of the gingival unit (slight change in color, little change in texture of any portion of the gingival unit), 2 = mild inflammation of the entire gingival unit, 3 = moderate inflammation (moderate change in texture, redness, edema and hypertrophy of the gingival unit, 4 = severe inflammation (marked redness and edema/hypertrophy, spontaneous bleeding or ulceration) of the gingival unit. Plaque indices were assessed for 6 surfaces on each tooth and the plaque index score was the average of the scores obtained from all teeth. The amount of plaque observed on each tooth was scored 0 to 3 as follows: 0 = no plaque notes; 1 = plaque seen only on the tip of an explorer passed over the tooth surface; 2 = plaque obvious with the naked eye; 3 = gross deposits of plaque present over the entire tooth.

### Systemic Health Selection Criteria/Assessment for Diabetes Status

For this study, diabetes status was established by self-reported history. Subjects who reported a history of diabetes were asked to confirm diabetic status by providing blood samples to measure HbA1c values. An individual was defined with Type 2 diabetes if hemoglobin A1C≥6.5%. Due to financial limitations, HbA1c levels were not assessed for those not reporting a history of diabetes. As a recent study suggested, the prevalence of diabetes (as measured by HbA1c>6.4) is likely low (extrapolated to be 5.0% with diabetes and 9.5% with pre-diabetes) in dental patients unaware of their diabetes status [48], we assumed that the prevalence of diabetes in the non-diabetes subject in this study would be similar and therefore not compromise the analysis.

### Saliva and Plasma Sample Collection

Saliva was collected in 50 mL polypropylene tubes and plasma was collected in Vacutainer tubes and anti-coagulated with EDTA. Samples were immediately frozen in a dry ice bath and stored at −80°C.

### Metabolomic Profiling Technology

Metabolomic profiling was performed as described previously [12], [49]. In summary, after the extraction of metabolites from each sample, the extracts were analyzed by GC/MS and LC/MS. We carried out chromatographic separation, followed by full-scan mass spectroscopy, to record and quantify all detectable ions presented in the samples [50]. We identified metabolites with known chemical structure by matching the ions' chromatographic retention index and mass spectral fragmentation signatures with reference library entries created from authentic standard metabolites under the identical analytical procedure as the experimental samples [51]. For ions that were not covered by the standards, additional library entries were added based on their unique ion signatures (chromatographic and mass spectral). After this, these ions could be routinely detected and quantified.

### Statistical Analysis

After normalization and imputation, the data were log-transformed. We then performed ANOVA and *t* tests to compare data obtained from the healthy, gingivitis, and periodontitis samples. Multiple comparisons were accounted for with the false discovery (FDR) rate method, and each FDR was estimated by *q*-values.

## Results

A total of 174 subjects were enrolled in this study (Table 1). The mean age of the non-diabetic subjects tended to be younger than the diabetic subjects (Table 2). Diabetic subjects had a higher BMI for all groups (Table 2). HbA1C testing confirmed diabetic status of the subjects self-reporting as diabetic.

**Table 1:** Sample Size of Study Groups.

	Healthy	Gingivitis	Periodontal	Totals
**Diabetic**	28	27	26	**81**
**Non-Diabetic**	25	26	29	**80**
**Totals**	**53**	**53**	**55**	**161**

**Table 2:** Demographics of Study Subjects.

Healthy n = 53		
	Non DM	DM
**Total**	**28**	**25**
**Male**	9	14
**Female**	19	11
**Mean Age**	37.5 (15.2)	50.2 (11.0)
**Mean BMI**	26.1 (4.2)	33.6 (9.9)
**Mean HbA1C** *	no data	7.1 (1.1)

Gingivitis n = 53		
	Non DM	DM
**Total**	**27**	**26**
**Male**	11	10
**Female**	16	16
**Mean Age**	35.9 (12.0)	53.8 (7.5)
**Mean BMI**	26.9 (5.7)	33.1 (7.6)
**Mean HbA1C**	no data	7.2 (1.5)

Periodontal n = 55		
	Non DM	DM
**Total**	**26**	**29**
**Male**	11	19
**Female**	15	10
**Mean Age**	49.7 (10.8)	54.5 (6.8)
**Mean BMI**	30.3 (6.0)	32.8 (7.9)
**Mean HbA1C**	no data	7.7 (1.9)

*HbA1c testing was done on purported diabetic subjects only.

An untargeted metabolomic profiling approach was used to assess the chemical milieu of saliva and plasma. In total, 161 individual saliva samples were collected, 80 from subjects with diabetes and 81 from subjects without diabetes. We detected 475 and 772 metabolites in saliva and plasma, respectively, of which 370 and 430 could be mapped to known chemical structures (Tables S1 and S2).

### Characterization of Diabetic and Non-Diabetic Samples

Welch's two sample *t* tests and ANOVA identified statistically significant differences in these saliva samples between non-diabetic subjects and those with diabetes. Approximately 14% of the detected metabolites (69 of 475) in saliva showed altered levels (*p*≤0.05) among the healthy samples when compared to the diabetic cohort, suggesting that these may be markers of diabetic status in humans (Table 3). Alternatively, comparison of the plasma from the same subjects resulted in 174 statistically significant changes. ANOVA analysis with either matrix did not produce a list of metabolites largely different from the *t* tests (data not shown).

**Table 3:** Overall statistical analysis of saliva and plasma samples from the healthy periodontal cohorts.

Summary of Altered Metabolites	*t*-Test Saliva (Diabetic vs. Non-diabetic)	*t*-Test Plasma (Diabetic vs. Non-diabetic)
Total number of comparisons *p*≤0.05	69	174
Biochemical direction of change	66 ↑/3 ↓*	115 ↑/59 ↓**

*66 metabolites were increased in saliva from subjects with diabetes, 3 metabolites were increased in saliva from non-diabetic subjects.

**115 metabolites were increased in plasma from subjects with diabetes, 59 metabolites were increased in plasma from non-diabetic subjects.

Glycemic control can be monitored by changes in 1,5-anhydroglucitol (1,5-AG). 1,5-AG is reabsorbed in the renal tubules, a process competitively inhibited by glucosuria, which leads to a reduction in its level in plasma. Thus, the greater the degree of glucosuria, the lower the amount of 1,5-AG present in the plasma [52]–[54]. The correlation between this reduction and the amount of glucose present in urine is so close that 1,5-AG represents a sensitive, real-time marker of glycemic control [55]. Thus, elevations of 1,5-AG in plasma are highly indicative of reduced glucosuria as a consequence of improved glycemic control. Not surprisingly, subjects with diabetes had significantly lower 1,5-AG and significantly higher levels of glucose in their plasma (Table 4). Additionally, diabetic subjects had significantly lower 1,5-AG in their saliva for all periodontal disease groups and higher levels of glucose in their saliva (Table 4). These observations lend confidence to the appropriateness of the control group, which was based on self-report of diabetes. None of the non-diabetic subjects had metformin in their plasma or saliva (data not shown).

**Table 4:** Relative levels of biochemicals linked to diabetic status in saliva and plasma*.

Matrix	Biochemical Name	Fold Change (Diabetc/Non-diabetic)	P value	Q value
Saliva	1-5 anhydroglucitol (1,5-AG)	0.93	0.0417	0.3041
	Glucose	2.66	0.0074	0.2228
	α-hydroxybutyrate (AHB)	2.14	0.0009	0.0958
Plasma	1-5 anhydroglucitol (1,5-AG)	0.61	0.0007	0.0168
	Glucose	1.35	9.46×10^−6^	0.0009
	α-hydroxybutyrate (AHB)	1.59	0.0012	0.0255

*Metformin was not detected in any of the non-diabetic samples.

α-hydroxybutyrate (AHB) is an early marker for insulin resistance in humans [52] as it arises from states of lactic and ketoacidosis or disrupted energy metabolism [56]–[61]. AHB levels were increased significantly in the diabetic cohort in plasma (Table 4) and were increased significantly in saliva in the healthy and gingivitis cohorts (data not shown). This may be a reflection of the increased energetic stress induced by the infection during the development of periodontitis or a translation of insulin resistance markers to saliva.

### High-Level Characterization of Diabetic and Non-Diabetic Periodontal Cohorts

The analysis of each periodontal cohort (diabetic and non-diabetic) separately resulted in the identification of several metabolites that were associated with the progression of gingival status (Table 5). The most striking find in the study was the direction of change in the periodontitis samples in the diabetic cohort. In the non-diabetic cohort, metabolites increased in abundance through disease progression, whereas many biochemicals increased in abundance when comparing the healthy to gingivitis groups, but then decreased in abundance in the diabetic subjects with periodontal disease suggesting an interaction between the diabetes signature and the periodontal signature.

**Table 5:** Overall statistical analysis of saliva samples comparing periodontal cohorts from diabetics and non-diabetics.

Summary of Altered Biochemicals	*t*-Test Saliva (Diabetic Gingivitis vs. Diabetic Healthy)	*t*-Test Saliva (Diabetic Periodontitis vs. Diabetic Healthy)	*t*-Test Saliva (Non-diabetic gingivitis vs. Non-diabetic Healthy)	*t*-Test Saliva (Non-diabetic periodontitis vs. Non-diabetic Healthy)
Total number of comparisons *p*≤0.05	35	54	20	64
Biochemical direction of change	25 ↑/10 ↓*	12 ↑/42 ↓**	17 ↑/3 ↓***	62 ↑/2 ↓****

*25 metabolites were increased in saliva from subjects with gingivitis, 10 metabolites were increased in saliva from diabetic, non-gingivitis subjects.

**12 metabolites were increased in saliva from diabetic subjects with periodontitis, 42 metabolites were increased in saliva from diabetic, periodontally healthy subjects.

***17 metabolites were increased in saliva from non-diabetic gingivitis subjects without diabetes, 3 metabolites were increased in saliva from non-diabetic, periodontally healthy subjects.

****62 metabolites were increased in saliva from non-diabetic periodontitis subjects, 2 metabolites were increased in saliva from non-diabetic, periodontally healthy subjects.

Both periodontal status and diabetes status of the subjects were major driving factors in the biochemical changes observed. For the majority of metabolites with altered concentrations in the non-diabetic cohort, the levels at gingivitis samples were found to be intermediate between those levels found in healthy and periodontitis samples (Table S1). This suggests that the metabolic changes induced by gingivitis are a continuum of those of periodontitis.

### Analysis of the Non-Diabetic Saliva Samples

Barnes et al. found differences in the biochemical signatures in saliva between non-diabetic subjects with healthy gingiva or periodontitis [12]. This report showed increased levels of metabolites in the saliva of the periodontal population reflecting enhanced degradation of macromolecules, including proteins, lipids, nucleotides and polysaccharides. Comparison of the non-diabetic cohort from the current study with these previous studies showed similar results (Table 6). This metabolic signature, which includes elevated levels of ω-6 fatty acids, is indicative of a hyper-inflammatory environment resulting from chronic bacterial infection and further support for increased macromolecular degradation of the periodontal tissues resulting from inflammation. Several metabolic signatures supported the notion that a more diverse and active microbial flora accompanied the inflammatory response resulting in gingivitis and periodontitis. Aromatic amino acid metabolites likely derived from bacteria were increased in periodontitis samples (Table 6). Moreover, carnitine, which is often the sole source of carbon and nitrogen for bacteria, was also elevated. Periodontal saliva samples also contained elevated levels of 3-dehydrocarnitine, a bacterial degradation product of carnitine, further supporting the utilization of carnitine in oral bacterial metabolism.

**Table 6:** Comparison of biochemical changes to previously published results.

Biochemical Pathway/Name	Barnes et al., J. Dent. Res. [12] Saliva Periodontitis vs. Healthy	Current Study Saliva (Non-diabetic subjects only) Periodontitis vs. Healthy
Purine Degradation (Link to Oxidative Stress)	Increased*	Increased
Dipeptides (Macromolecular degradation of proteins)	Increased	Increased
Amino Acid Metabolites (p-cresol sulfate, Bacterial)	Increased	Increased
Carbohydrates (monosaccharides indicative of amylase activity)	Increased	Increased
Energy Metabolites (TCA cycle, indicative of energetic stress)	Unchanged	Increased
Uridine (DNA/RNA Degradation)	Increased	Increasing trend**
Allantoin	Increased	Increasing trend
ω-6 fatty acids (link to inflammation)	Increased	Increased
Fatty Acids	Increased	Increased
Acetylcarnitine	Increased	Increased
Carnitine	Increased	Unchanged
3-dehydrocarnitine	Increased	Increased

*Increased with statistically significant differences (*p*≤0.05) in biochemicals from those pathways. I

**Increasing trend reflects differences (0.05<*p*≤0.10) in biochemicals from those pathways.

### Comparison of Biochemical Signatures in Saliva Samples from Diabetics vs Non-Diabetic subjects

#### Oxidative Stress and Anti-Oxidant Capacity

Purine degradation is regulated, in part, by oxidative stress [62], and increased purine degradation serves as an indication of an increased inflammatory response. Specifically, the conversion of hypoxanthine to xanthine and the subsequent conversion of xanthine to uric acid require O_2_ to activate xanthine oxidase [63], [64] and these reactions are coupled with a reduction of oxygen to generate superoxides in the form of O_2_^-^ and H_2_O_2_. Urate is known to function in redox balance as a radical scavenger or antioxidant under conditions of oxidative stress and in the process converted to other products (e.g. allantoin). In the context of gingival disease, increased oxidative stress would result in higher levels of purine degradation metabolites, reflected in increased levels of hypoxanthine and xanthine. The results of this study are consistent with the results from previous studies [11], [37]. Metabolites in the purine degradation pathway were statistically elevated in the diabetic cohort compared to the non-diabetic cohort (Table 7).

**Table 7:** Relative levels of biochemicals linked to purine degradation and anti-oxidant status in saliva samples from diabetics.

Biochemical Name	Fold Change (Diabetic -Gingivitis/Diabetic - Healthy)	P value	Q value
Adenosine	1.37	0.0943	0.6881
Inosine	1.70	0.0294	0.629
Guanine	2.40	0.0673	0.6546
Guanosine	2.06	0.0343	0.629
Xanthine	1.49	0.0434	0.629
Glutathione, oxidized (GSSG)	1.40	0.07	0.6546
Cysteine-glutathione disulfide	1.57	0.061	0.6444

The levels of cysteine-glutathione disulfide and oxidized glutathione increased with gingival disease in non-diabetics and diabetics (Table 7). Glutathione plays a central role in the cellular defense against free radicals and xenobiotics. The increased levels of these oxidized glutathione species in saliva indicated increased oxidative stress.

#### Lipid and Sphingolipid Metabolism

Several lipid inflammatory mediators increased significantly in saliva from both the non-diabetic and diabetic groups during periodontal disease progression (Table 8). In this state of oxidative stress, lipoxygenase enzymes oxidize fatty acids to generate lipid signaling molecules such as 12-HETE. In saliva, arachidonate and 12-HETE increased in the non-diabetic cohort with periodontal disease and also increased in the diabetic gingivitis cohort compared to the healthy group, but was reduced in periodontitis (Table 8). Sphingomyelin, ceramides and glycosphingolipids are molecules that reside in the plasma membrane and participate in intracellular signaling cascades. Palmitoyl sphingomyelin was elevated in saliva of diabetic and non-diabetic subjects periodontitis (Table 8).

**Table 8:** Fatty acids and sphingomyelins inreased in saliva from subjects with diabetes and periodontal disease.

Biochemical Name	Fold Change (Diabetic - Gingivitis/Diabetic - Healthy)	P value	Q value
12-HETE	2.41	0.0127	0.629
Linoleate (18∶2n6)	3.22	0.0269	0.629
Linolenate [α or γ; (18∶3n3 or 6)]	4.52	0.0476	0.629
Docosapentaenoate (n3 DPA; 22∶5n3)	1.63	0.0483	0.629
Arachidonate (20∶4n6)	2.00	0.0631	0.6517
Palmitoyl sphingomyelin	1.65	0.0422	0.629

## Discussion

We used metabolomic profiling technology to analyze saliva from subjects with and without diabetes who had a healthy gingiva, gingivitis, or periodontitis. In addition to verifying previously published observations in saliva [12] and GCF [11], [37], we also identified in the present study salivary biochemical changes associated with diabetes, as well as changes associated with periodontal disease. The results from this analysis show that human diabetic subjects with periodontal disease have an increased purine degradation signature, a decreased redox balance capacity (Table 7) and altered ω-3/ω-6 fatty acid profiles (Table 8) in saliva.

Redox balance is vital for maintaining cellular homeostasis. Oxidative stress, caused by over production of reactive oxygen species and insufficient cellular anti-oxidant capabilities, can lead to cell damage. Glutathione and other anti-oxidant molecules serve to protect cellular proteins and structures from oxidative stress caused by reactive oxygen species. Glutathione is a thiol-containing tripeptide that plays a central role in cellular defense against free radicals and xenobiotics [65]–[68] and is the major intracellular redox mediator and it is pivotal for controlling the electrical or redox gradient across the mitochondrial inner-membrane [69], [70]. Glutathione in the mitochondrial intermembrane space plays a intricate role in the mechanism of redox balance in living cells through its linkage to the redox milieu in the cytosol [71]. Oral interventions that enhance redox balance have been shown to be effective at relieving oral symptoms. For example, Hansen and Grunnet showed that taurine plays a significant role in mitochondria in pH buffering that not only affects the status of glutathione, but also the NADH/NAD+ redox pair [72]. These molecules were all found to be elevated in saliva from the diabetic subjects.

Several lipid compounds are important for the initiation and propagation of inflammatory signaling in cells and tissues. Linoleic acid and arachidonic acid give rise to many of these metabolites. These lipid signaling inflammatory mediators are liberated from the plasma membrane phospholipids by phospholipase A_2_ enzymes ultimately resulting in the production of linoleic and arachidonic acid-derived eicosanoids which help to mediate the inflammatory response [73]. Arachidonic acid release can also be initiated by collagen, thrombin, bradykinin, serotonin, and adrenaline, which increase in tissues during tissue damage and the early stages in inflammation and oxidative stress. Immune cells such as macrophages and neutrophils produce reactive oxygen species and reactive nitrogen species during cell activation that have the potential to damage lipids, proteins, and other molecules. Interestingly, these compounds were found to be elevated in the saliva of diabetic subjects with gingivitis.

Mobilization of lipid signaling pathways results in the synthesis of ceramides or the liberation of ceramides from intracellular stores. The activity of ceramidases results in the formation of sphingosine and sphingomyelin molecules that function in an array of intracellular signaling or maintenance of cell membrane architecture including apoptosis [74], [75]. Depending on the specific cellular type and mechanism of activation, sphingomyelins and ceramides can serve activation or inhibitory purposes [76]. The formation of ceramides and sphingomyelins has been linked to cell death through nutrient starvation [74]–[76] as well as an important signaling molecule for cell differentiation and polarity [77]. *Porphyromonas gingivalis* is known to induce and alter sphingomyelin and ceramide signaling during infection [78].

Prediction of risk and accurate diagnosis of current disease activity may facilitate effective prevention and treatment of gingival diseases. GCF and saliva have been extensively examined in attempts to assess the oral disease status. The markers identified in this study may be leveraged to monitor the effectiveness of interventions as well as identify the mechanisms of action for these interventions. Several different approaches have confirmed that identifying a single marker characteristic of a disease is unlikely; rather, a combination of biomarkers would likely constitute an effective clinical test. For example, insulin resistance is a feature if the pre-diabetes stage in humans prior to the development of diabetes. Untargeted biochemical profiling to identify biomarkers of insulin resistance following the development of targeted assays and validation of the targeted assays in a clinical cohort resulted in the development of a diagnostic that monitors insulin sensitivity status in humans [52], [79]. By monitoring selected metabolites representing the diverse biochemical pathways, it is therefore possible to identify unique biomarkers and to monitor during the course of periodontal treatment in diabetics. In conclusion, this study demonstrated that biochemical profiling technology is a powerful tool for dental research and the results provide new mechanistic insights and treatment strategies that could be specific for oral health of diabetics. Additionally, the biochemicals identified in this study that segregated the individuals by periodontal status could be leveraged or developed into a rapid diagnostic to monitor periodontal disease activity.

## Supporting Information

Table S1 **Saliva Metabolic Profiling and Pathway Analysis.** Statistical analyses for each biochemical detected in saliva between each of the gingival and diabetic cohorts.
(XLSX)

Table S2 **Plasma Metabolic Profiling and Pathway Analysis.** Statistical analyses for each biochemical detected in plasma between each of the gingival and diabetic cohorts.
(XLSX)
