# Correction: Global Metabolomic Analysis of Human Saliva and Plasma from Healthy and Diabetic Subjects, with and without Periodontal Disease

**DOI:** 10.1371/journal.pone.0114091

**Published:** 2014-11-20

**Authors:** The PLOS ONE Staff

There are errors in the author affiliations. The affiliations should appear as shown here:

Virginia M. Barnes^1^, Adam D. Kennedy^2^, Fotinos Panagakos^1^, William Devizio^1^, Harsh M. Trivedi^1^, Thomas Jönsson^2^, Lining Guo^2^, Shannon Cervi^3^, Frank A. Scannapieco^3^*

1 Colgate Palmolive Technology Center, Piscataway, NJ, United States of America

2 Metabolon, Durham, NC, United States of America

3 Department of Oral Biology, School of Dental Medicine, University at Buffalo, State University of New York, Buffalo, NY, United States of America
